# Nachwuchsförderung in der Chirurgie: Laparoskopie in der Vorklinik – zu früh oder genau richtig?

**DOI:** 10.1007/s00104-024-02145-3

**Published:** 2024-08-12

**Authors:** Marvin Heimke, Tillmann Heinze, Jorun Baumann, Bodo Kurz, Ibrahim Alkatout, Thilo Wedel

**Affiliations:** 1 Anatomisches Institut, Zentrum für Klinische Anatomie, Kurt-Semm-Zentrum für laparoskopische und roboterassistierte Chirurgie, Christian-Albrechts-Universität zu Kiel, Otto-Hahn-Platz 8, 24118 Kiel, Deutschland; 2 Klinik für Gynäkologie und Geburtshilfe, Kurt-Semm-Zentrum für laparoskopische und roboterassistierte Chirurgie, Universitätsklinikum Schleswig-Holstein Campus Kiel, Kiel, Deutschland

**Keywords:** Lehrprojekt, Chirurgische Karriere, Nachwuchsförderung, Laparoskopie, Anatomie, Teaching project, Surgical career, Young talent recruitment, Laparoscopy, Anatomy

## Abstract

**Hintergrund:**

Aufgrund eines abnehmenden Interesses unter Medizinstudierenden an einer chirurgischen Laufbahn, der Auswirkungen des demographischen Wandels sowie der technischen Herausforderungen besteht ein zunehmender Bedarf an gezielter Nachwuchsförderung in der Chirurgie. Vor diesem Hintergrund wurde ein Lehrprojekt entwickelt, welches Medizinstudierende bereits in der Vorklinik mit minimal-invasiven Techniken der Chirurgie vertraut machen und so das Interesse an operativen Fachbereichen steigern soll.

**Methodik:**

Im Rahmen des regulären vorklinischen anatomischen Präparierkurses wurden folgende Module zur laparoskopischen Chirurgie integriert: (1) klinische Vorlesung zur Technik und Einsatz der Laparoskopie, (2) explorative Live-Laparoskopie an einem Körperspender, (3) praktische Übungen an Laparoskopietrainern. Die Effekte dieses Lehrprojekts auf das Interesse an einer chirurgischen Laufbahn und das klinisch-anatomische Verständnis wurden von 295 Medizinstudierenden evaluiert.

**Ergebnisse:**

Die Evaluation zeigte ein ausgeprägtes, geschlechtsunabhängiges Interesse, chirurgische Fertigkeiten bereits in der Vorklinik zu erlernen. Das Lehrprojekt führte zu einer signifikanten Steigerung des Interesses an einer chirurgischen Laufbahn. Zudem förderte die Einbindung der laparoskopischen Lehrmodule in den vorklinischen Anatomieunterricht die Lernmotivation und das Verständnis für die klinisch relevante topographische Anatomie.

**Diskussion:**

Die Integration praxisnaher chirurgischer Inhalte in die vorklinische anatomische Lehre kann die Attraktivität chirurgischer Disziplinen erhöhen und gleichzeitig die anatomische Lehre optimieren. Längsschnittstudien sind erforderlich, um die Nachhaltigkeit dieser frühzeitigen klinischen Lehrmodule auf die Karriereentscheidung von Medizinstudierenden zu untersuchen.

**Zusatzmaterial online:**

Zusätzliche Informationen sind in der Online-Version dieses Artikels (10.1007/s00104-024-02145-3) enthalten.

## Abstract (en)

**Background:**

Due to the declining interest among medical students in pursuing a surgical career, the impact of demographic changes and the technical challenges, there is an increasing need to attract medical students to the surgical profession. Against this backdrop, a teaching project was developed to familiarize medical students with minimally invasive surgical techniques early in the preclinical education, thereby increasing the interest in surgery.

**Material and methods:**

Within the framework of the regular anatomical dissection course the following modules on laparoscopic surgery were integrated: (1) clinical lecture on the technique and application of laparoscopy, (2) exploratory live laparoscopy on a body donor and (3) practical exercises on laparoscopic trainers. The impact of this teaching project on the interest in a surgical career and on the clinical anatomical understanding was evaluated among 295 participating medical students.

**Results:**

The evaluation revealed a pronounced gender-independent interest in learning surgical skills early in the preclinical study phase. The teaching project led to a significantly increased interest in pursuing a surgical career. Moreover, the incorporation of laparoscopic teaching modules into the preclinical anatomy course enhanced the learning motivation and understanding of clinically relevant topographic anatomy.

**Conclusion:**

The integration of practical surgical content into preclinical anatomical education can increase the attractiveness of surgical disciplines and simultaneously optimize teaching of anatomy. Longitudinal studies are required to examine the sustainability of these clinical teaching modules in career decisions of medical students.

## Hintergrund

In den letzten Jahren ist sowohl in Deutschland als auch international ein sinkendes Interesse von Medizinstudierenden an einer chirurgischen Karriere zu verzeichnen [10]. Aktuelle Studien zeigen, dass die Zahl der Bewerberinnen und Bewerber für eine Facharztweiterbildung in der Allgemeinchirurgie rückläufig ist [25, 26]. In einer Umfrage unter 715 Chef- und (Leitenden) Oberärztinnen und -ärzten deutscher chirurgischer Kliniken gaben 80 % der Teilnehmenden sowohl einen numerischen als auch qualitativen Bewerbermangel an. In einigen Kliniken blieben vakante Stellen teilweise über Monate unbesetzt [26].

Gleichzeitig sind wir mit einer zunehmenden Überalterung der stationär tätigen Chirurginnen und Chirurgen und deren baldigem Ausscheiden aus dem Berufsleben konfrontiert. Die Statistik der Bundesärztekammer ergab hierzu einen Anstieg des Anteils der über 60-Jährigen von 10,7 % (2015) auf 14,1 % (2021; [8]). Es wird davon ausgegangen, dass im Jahr 2030 nahezu jede vierte Facharztstelle in der Chirurgie unbesetzt sein wird [14]. Zudem bedingt der demographische Wandel mit einer zunehmenden Alterung der Gesellschaft statistisch gesehen einen steigenden Bedarf an stationär chirurgischer Versorgung [12]. Darüber hinaus werden die technischen Herausforderungen durch die minimal-invasive Chirurgie für den chirurgischen Nachwuchs zunehmend komplexer [1, 2].

In Anbetracht dieser Fakten sind zahlreiche Initiativen entstanden, um das Interesse von Medizinstudierenden an einer chirurgischen Karriere durch gezielte Trainingsmaßnahmen und Praktika zu fördern [4, 5, 7, 11, 15, 19, 23, 25]. Allerdings zielen viele dieser Maßnahmen vorwiegend auf fortgeschrittene Studienabschnitte ab [21]. Dabei ist bekannt, dass das Interesse für die Chirurgie insbesondere zu Beginn des Studiums ausgesprochen hoch ist und sich erst im Verlauf des Studiums verliert [12, 18]. In diesem Zusammenhang wird kritisiert, dass die chirurgischen Fachgesellschaften ihre „Startvorteile“ gegenüber anderen Fachdisziplinen im Rahmen der Nachwuchsakquise nicht ausreichend ausschöpfen [21].

Vor diesem Hintergrund wurde im Rahmen einer vorklinisch-klinischen Kooperation zwischen dem Anatomischen Institut und der Klinik für Gynäkologie und Geburtshilfe der Universität Kiel ein Lehrprojekt etabliert, welches Medizinstudierende bereits in der Vorklinik mit den Prinzipien und Techniken der minimal-invasiven Chirurgie vertraut macht. Das Konzept einer frühzeitigen Konfrontation mit modernen Operationstechniken sowie einer Verknüpfung anatomischer Fakten mit praxisnahen, chirurgisch relevanten Szenarien wurde hinsichtlich folgender Effekte überprüft: Interessensteigerung an einem operativen Fachgebiet sowie Verbesserung der Lernmotivation und des Verständnisses der topographischen Anatomie.

## Methodik

### Integration des Lehrprojektes in den Anatomieunterricht

Das Lehrprojekt wurde in den Kursus für Makroskopische Anatomie (Präparierkurs) des Anatomischen Instituts der Medizinischen Fakultät der Universität Kiel integriert. In den Wintersemestern 2022/23 und 2023/24 wurde der zweiwöchige Kursabschnitt „Situs“, bei dem die großen Körperhöhlen präpariert werden, um theoretische und praktische Module zur minimal-invasiven Chirurgie erweitert. Dieses fakultative Angebot stand allen Teilnehmenden (*n* = 384) der beiden Präparierkurse zur Verfügung.

### Module des Lehrprojektes

**Abb. 1:**
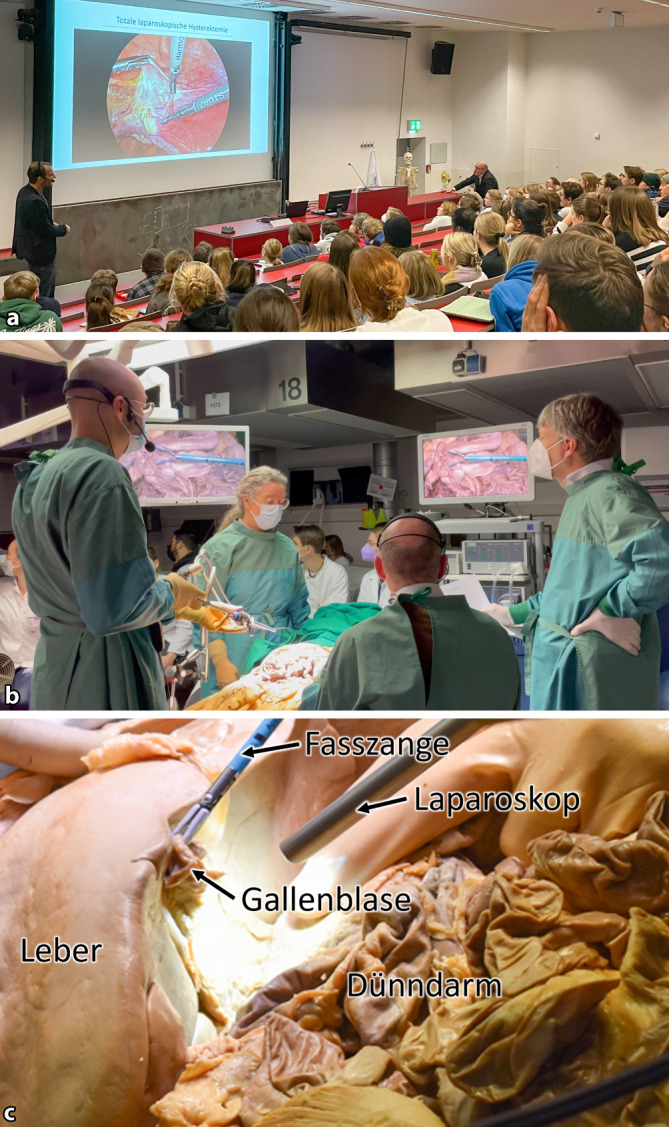
Lehrprojekt „Laparoskopie im Präparierkurs“. **a** Klinik-Vorlesung zur Entwicklung, Technik und Einsatz der Laparoskopie, **b** Livedemonstration einer explorativen Laparoskopie an einem Körperspender mit Erläuterung anatomischer Landmarken und Strukturen aus laparoskopischen Blickwinkeln, **c** laparoskopischer Blickwinkel in den rechten Oberbauch (Leberunterfläche und Gallenblase)

Das Lehrprojekt bestand aus drei aufeinander aufbauenden Modulen:

1. **Klinik-Vorlesung:** In dieser Lehrveranstaltung wurden die Medizinstudierenden über die Entwicklung, Techniken und Einsatz der Laparoskopie durch einen erfahrenen klinischen Referenten (I. A.) informiert und erhielten anhand ausgewählter Operationsvideos Einblicke in die praktische Durchführung der Laparoskopie. Die Vorteile und Limitationen bzw. Herausforderungen der minimal-invasiven Chirurgie wurden erörtert (Abb. 1a).
2. **Laparoskopische Situsexploration an einem Körperspender:** Im Anschluss wurde allen Medizinstudierenden im Präpariersaal eine explorative Live-Laparoskopie (Image 1 HD Kamera, HOPKINS Optik 30°, Image 1 Connect TC200, Image 1 H3-Link, SCB Power LED 175, Storz, Tuttlingen, Deutschland) des Abdominal- und Beckensitus an einem formalinfixierten Körperspender demonstriert (Abb. 1b). Die laparoskopische Exploration wurde regionenspezifisch durchgeführt, interaktiv kommentiert und auf Beamer und große Monitore übertragen. Der Fokus lag auf der Vermittlung chirurgisch relevanter anatomischer Landmarken und Strukturen. Im Gegensatz zu konventionellen Lehrbuch- und Atlasdarstellungen der Anatomie (Frontalansicht, aufrecht stehende Position) wurde die topographische Anatomie aus laparoskopisch etablierten Blickwinkeln präsentiert (Abb. 1c).
3. **Praktische Übungen am Laparoskopietrainer:** Für die praktischen Übungen wurden im Präpariersaal zwei Laparoskopietürme (3DV-190, CLV-S190, VC-190, Endoeye Flex 3D, Olympus, Tokyo, Japan) mit Laparoskopietrainern (Szabo Berci-Sackier, Storz, Tuttlingen, Deutschland bzw. Kroton Laparoscopic Trainer, Kroton Medical technology, Warszawa, Poland) und Laparoskopieinstrumenten (laparoskopische Zange CY010 und C4130, laparoskopische Schere CB030, Applied Medical, Düsseldorf, Deutschland; laparoskopische 5‑mm-Makro-Nadelhalter, Karl Storz, Tuttlingen, Deutschland) ausgestattet.

**Abb. 2:**
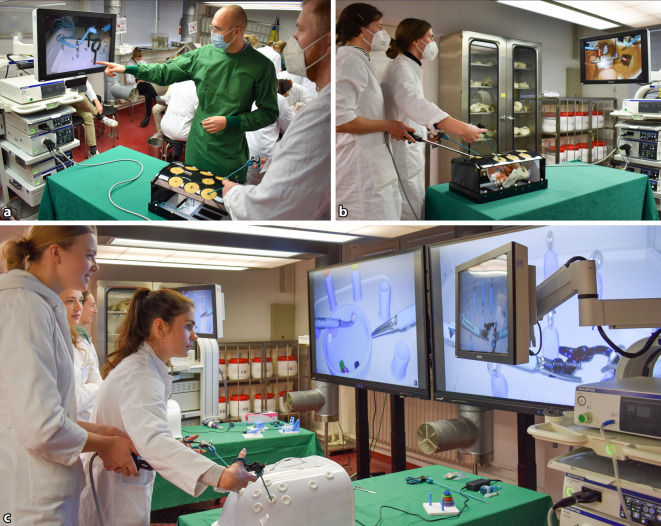
Laparoskopieübungen im Präpariersaal. **a** Einweisung in die Übung „Fadendurchzug durch Ösen“, **b** selbstständige Durchführung der Übung „Bügelperlen-Transfer“ in einem anatomischen Beckenmodell, **c** paralleles Training an zwei laparoskopischen Arbeitsstationen

Nach allgemeiner Einweisung in die Funktionsweise und Handhabung der laparoskopischen Arbeitsstationen erhielten alle Medizinstudierende ein individuelles 15-minütiges Zeitfenster während des Präparierkurses zur Durchführung laparoskopischer Übungen, die Bestandteil der validierten Laparoskopiekurskonzepte der Kiel School of Gynaecological Endoscopy sind (Abb. 2; [1, 2, 20]): (1) Transfer von Bügelperlen mittels Fasszangen zwischen zwei Halterungen in einem anatomischen Beckenmodell, (2) Führung und Durchzug eines Fadens durch 7 unterschiedlich positionierte Ösen.

Alle Übungen wurden durch laparoskopisch erfahrene Mitarbeiterinnen und Mitarbeiter sowie von studentischen Tutorinnen des klinischen Studienabschnittes angeleitet. Darüber hinaus standen freie Zeitfenster außerhalb des Präparierkurses für weitere Übungen zur Verfügung. Hier konnten die Medizinstudierenden auch erste Erfahrungen zu laparoskopischen Dissektionen und Nahttechniken an entsprechenden Simulationsmodellen sammeln.

### Evaluation des Lehrprojektes

Die Datenschutz-Grundverordnung(DSGVO)-konforme, anonymisierte und freiwillige Evaluation erfolgte im Anschluss an den Präparierkursabschnitt mittels eines Onlinefragebogens (LimeSurvey GmbH, Hamburg, Deutschland). Der Fragebogen beinhaltete 15 Fragen und gliederte sich in vier Abschnitte: (1) soziodemographische Daten, (2) Nutzung der Laparoskopietrainer, (3) Relevanz des Laparoskopietrainings für die anatomische Lehre, (4) Einfluss des Laparoskopietrainings auf das Interesse an einem operativen Fachbereich. Für jeden Abschnitt bestand die Möglichkeit zu Freitextkommentaren.

### Datenauswertung

Die Datenauswertung erfolgte mittels SPSS 29.0 (SPSS Inc., IBM Company, Armonk, NY, USA). Zur Überprüfung der Datensätze auf Normalverteilung wurde der Shapiro-Wilk-Test durchgeführt. Unterschiede im Antwortverhalten bezüglich der Variablen „Geschlecht“ und „vorangegangene Ausbildung/Studium“ wurden nach Erstellung einer Kontingenztafel mittels χ^2^-Test und anschließendem Bonferroni-Post-hoc-Test geprüft. Unterschiede zwischen dem Interesse an einer chirurgischen Weiterbildung vor und nach dem Lehrprojekt wurden durch den McNemar-Bowker-Test ermittelt. Die Tests waren zweiseitig. Das Signifikanzniveau betrug 5 %.

## Ergebnisse

### Soziodemographische Daten

Von 384 Medizinstudierenden, die während der Präparierkurse am Lehrprojekt teilnahmen, übersandten 295 eine vollständige Evaluation (77 % Rücklaufquote). Das Medianalter betrug 22,4 Jahre (18–40 Jahre), 68 % waren weiblich, 30 % männlich, 2 % gaben ihr Geschlecht als divers an oder machten hierzu keine Aussage (Tab. 1). Vor Beginn des Medizinstudiums hatten bereits 42 % der Teilnehmenden eine Ausbildung oder ein Studium begonnen bzw. abgeschlossen. Der Großteil der vorangegangenen Ausbildungen fand in medizinischen Berufsfeldern statt, z. B. Gesundheits- und Krankenpflege (37 %), Rettungsdienst (18 %), medizinische, radiologische, operativ-technische Assistenz (7 %) oder Physiotherapie (7 %; Tab. 2).

**Tab. 1:** Charakterisierung der Teilnehmenden am Lehrprojekt

Gesamtzahl der Teilnehmenden	384 Personen
Rücklaufquote Evaluation	76,8 % (295)
Medianalter	22,4 Jahre (18 bis 40 Jahre)
Geschlechterverteilung	67,5 % weiblich (199) 30,2 % männlich (89) 2,4 % divers oder keine Angabe (7)

**Tab. 2:** Berufsausbildung/Studium vor Medizinstudium

*Anteil mit Berufsausbildung/Studium*	
Gesamt	42,0 % (124)
Weiblich	41,2 % (82)
Männlich	43,8 % (39)
*Art der Berufsausbildung/Studium*	
Gesundheits- und Krankenpflege	37,1 % (46)
Studium	21,8 % (27)
Rettungsdienst	17,7 % (22)
ATA, MTA, MTLA, MTR	6,5 % (8)
Physiotherapie	6,5 % (8)
MFA	4,8 % (6)
OTA	3,2 % (4)
Sonstiges	2,4 % (3)

*OTA* Operationstechnische Assistenz, *ATA* Anästhesietechnische Assistenz, *MTLA* Medizinisch-technische Laboratoriumsassistenz, *MTA* Medizinisch-technische Assistenz, *MTR* Medizinische*r Technolog*in für Radiologie, *MFA* Medizinische*r Fachangestellte*r; *Studium* Bioinformatik, Biologie, Industriedesign, Ingenieurswissenschaft, Kognitionswissenschaft, Maschinenbau, Pharmazie, Physik, Psychologie, Soziologie, Veterinärmedizin, Wirtschaft, Zahnmedizin

### Nutzung der Laparoskopietrainer

Der Großteil der Medizinstudierenden nutzte die Laparoskopietrainer während des zweiwöchigen Abschnitts für 10–30 min (57 %) oder 30–60 min (25 %). Bei einer Nutzungsdauer von unter 10 min (9 %) wurden als Gründe am häufigsten eine „*zu hohe Nachfrage durch andere Studierende*“ (50 %) sowie „*mangelnde Zeit aufgrund des arbeits- und lernintensiven Präparierkurses*“ (42 %) angegeben. 98 % der Teilnehmenden hielten den Zeitpunkt für angemessen, bereits während des Präparierkurses mit der minimal-invasiven Chirurgie vertraut gemacht zu werden. In Freitextkommentaren wurde betont, dass die Verknüpfung vorklinisch-theoretischer Lerninhalte mit chirurgisch-praktischen Anwendungen zu einer deutlich gesteigerten Lernmotivation führte. Gleichzeitig sei die spielerische Annährung an die minimal-invasive Chirurgie eine willkommene Abwechslung zum theoretischen Faktenlernen gewesen.

### Relevanz des Laparoskopietrainings für die anatomische Lehre

**Abb. 3:**
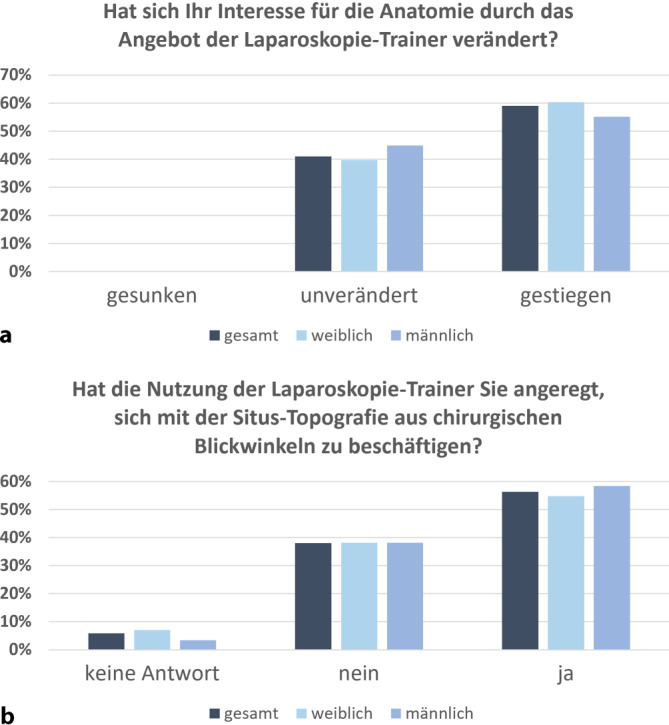
Relevanz des Laparoskopietrainings für die anatomische Lehre. **a** Interesse für Anatomie, **b** Auseinandersetzung mit der topographischen Anatomie aus chirurgischen Blickwinkeln. Der geschlechtsspezifische Vergleich zeigt keine signifikanten Unterschiede

Über die Hälfte der Medizinstudierenden (59 %) gaben an, dass ihr Interesse am Erlernen der Anatomie durch die laparoskopischen Übungen und die damit verbundenen klinischen Zusammenhänge und Anwendungen gestiegen sei (Abb. 3a). 56 % der Teilnehmenden äußerten, dass das laparoskopische Training sie animiert habe, sich vermehrt mit der topographischen Anatomie aus dem Blickwinkel des Operateurs auseinanderzusetzen (Abb. 3b). Dazu wurde in Freitextkommentaren der erzielte Erkenntnisgewinn hinsichtlich der Lagebeziehungen der Organe sowie klinisch relevanter anatomischer Landmarken hervorgehoben. Einige Teilnehmende merkten jedoch an, dass das laparoskopische Training für sie lediglich die Geschicklichkeit sowie die visuell-räumliche Wahrnehmung geschult habe. Um eine intensivere Auseinandersetzung mit der chirurgischen Anatomie zu fördern, wurde angeregt, die Laparoskopietrainer mit realistischen anatomischen Modellpräparaten auszustatten.

### Einfluss des Laparoskopietrainings auf das Interesse an chirurgischen Fertigkeiten und einem operativen Fachbereich

**Abb. 4:**
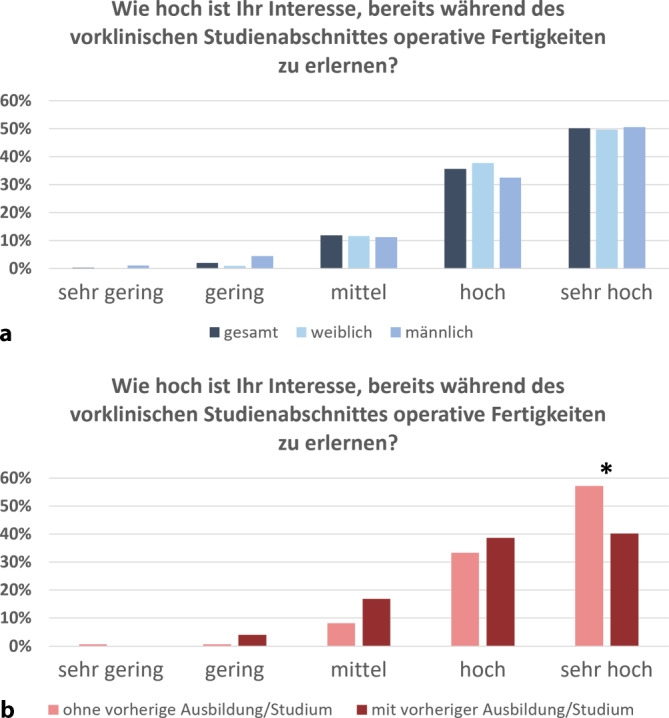
Interesse am Erlernen operativer Fertigkeiten im vorklinischen Studienabschnitt. **a** Der geschlechtsspezifische Vergleich zeigt keinen signifikanten Unterschied, **b** das Antwortverhalten zwischen den Kohorten ohne und mit Ausbildung/Studium unterschied sich signifikant (* *p* < 0,01)

Das Interesse der Medizinstudierenden, bereits während des vorklinischen Studienabschnittes erste chirurgische Fertigkeiten zu erwerben, wurde als hoch (36 %) bzw. sehr hoch (50 %) eingestuft (Abb. 4a). In Freitextkommentaren brachte die Mehrheit zum Ausdruck, dass sie so früh wie möglich praktische Fertigkeiten erlernen möchte. Als wesentlicher Grund wurde hier angeführt, dass die Verknüpfung von Theorie und Praxis einen höheren Anreiz schaffe, auch komplexe anatomische Sachverhalte zu lernen. Zudem führe das Einüben praktischer chirurgischer Fertigkeiten zu einer Identifikation mit der zukünftigen ärztlichen Berufsrolle und somit zu einer generell gesteigerten Motivation für das Medizinstudium. So hätten sich einerseits viele Medizinstudierende mehr Zeit zum Üben an den Laparoskopietrainern gewünscht. Andererseits wurde auch zu bedenken gegeben, dass aufgrund des umfangreichen Lehrplans eine Trennung von vorklinischen und klinischen Lehrinhalten grundsätzlich gerechtfertigt sei.

**Abb. 5:**
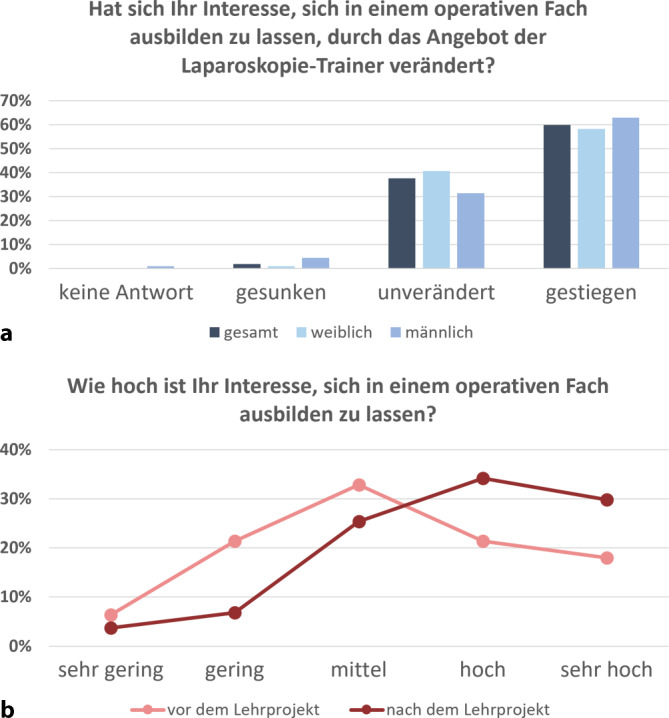
Relevanz des Laparoskopietrainings für das Interesse an einer Ausbildung in einem chirurgischen Fachbereich. **a** Veränderung des Interesses an chirurgischem Fachbereich durch Nutzung des Laparoskopietrainers, **b** Interesse an chirurgischem Fachbereich vor und nach dem Lehrprojekt. Der geschlechtsspezifische Vergleich zeigt keine signifikanten Unterschiede

Annähernd zwei Drittel (60 %) der Teilnehmenden gaben an, dass ihr Interesse an einer Ausbildung in einem chirurgischen Fachgebiet infolge des laparoskopischen Trainings gestiegen sei, lediglich 2 % äußerten ein gesunkenes Interesse (Abb. 5a). So bekundete die größte Kohorte der Medizinstudierenden (33 %) vor dem Lehrprojekt ein „mittleres Interesse“ an einer Karriere in einem operativen Fach (Abb. 5b). Nach Abschluss des Lehrprojekts hingegen änderte sich die Einschätzung signifikant: Hier gaben die größten Kohorten ein „hohes Interesse“ (34 %) bzw. ein „sehr hohes Interesse“ (30 %) an.

**Abb. 6:**
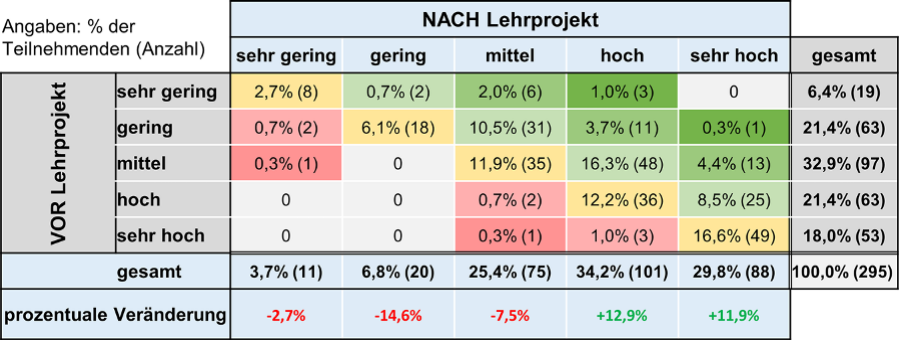
Kontingenztafel zum Interesse an chirurgischer Weiterbildung vor und nach dem Lehrprojekt. In jedem Feld ist der prozentuale und absolute Anteil der Studierenden angegeben, deren Interesse an einer chirurgischen Weiterbildung sich nach dem Lehrprojekt im Vergleich zum Ausgangsniveau verändert hat (*grüne* Felder: Steigerung des Interesses; *rote* Felder: Verringerung des Interesses) oder gleichgeblieben ist (*gelbe* Felder). 47,5 % der Studierenden gaben eine Steigerung ihres Interesses an (Summe aller *grünen* Felder), 49,5 % der Studierenden gaben keine Veränderung an (Summe aller *gelben* Felder), 3,1 % der Studierenden gaben eine Verringerung ihres Interesses an (Summe aller *roten* Felder). Die Veränderung im Antwortverhalten war signifikant (McNemar-Bowker-Test, *p* < 0,001)

In Freitextkommentaren berichteten einige Medizinstudierende, dass ihre anfänglichen Zweifel hinsichtlich ihrer handwerklichen Fähigkeiten und der damit verbundenen Eignung für eine chirurgische Tätigkeit ausgeräumt wurden, sodass sie nun eine Ausbildung in einem operativen Fach durchaus in Betracht ziehen würden. Dies spiegelt sich u. a. darin wider, dass durch das Lehrprojekt das Interesse einiger Studierender an einer zukünftigen chirurgischen Tätigkeit von „sehr niedrig“ auf „mittel“ oder sogar „hoch“, und in einigen Fällen von „niedrig“ auf „hoch“ oder „sehr hoch“ gestiegen war – was einer Interessenzunahme um zwei bis drei Stufen entspricht (Abb. 6). Demgegenüber wurde nur bei einer geringen Anzahl von Studierenden eine Abnahme des ursprünglich hohen Interesses beobachtet.

### Einfluss des Geschlechts und vorangegangener Ausbildung/Studium

Die Evaluation des Lehrprojektes ergab keine relevanten geschlechtsspezifischen Unterschiede im Antwortverhalten. Lediglich zur Frage, ob sich das anatomische Verständnis durch das Angebot des Laparoskopietrainings verändert habe, gaben 37 % der Frauen und nur 25 % der Männer ein gestiegenes anatomisches Verständnis durch das Lehrmodul an (Daten nicht gezeigt). Teilnehmende, die bereits eine Ausbildung oder ein anderes Studium vor dem Medizinstudium begonnen hatten, zeigten ein geringeres Interesse am Erlernen operativer Techniken im vorklinischen Studienabschnitt im Vergleich zu denen ohne vorherige Ausbildung/Studium – wobei dennoch 79 % ein „hohes“ bis „sehr hohes“ Interesse äußerten (Abb. 4b). Ebenso war auch der Wunsch nach einer Weiterbildung in einem operativen Fach nach dem Lehrmodul signifikant geringer als bei Medizinstudierenden ohne vorherige Ausbildung. Unabhängig davon ergab sich jedoch auch im Kollektiv mit vorheriger Ausbildung bei 50 % eine Steigerung des Interesses an einer chirurgischen Weiterbildung (Daten nicht gezeigt).

## Diskussion

Im vorgestellten Lehrprojekt wurde untersucht, ob eine frühzeitige Vorstellung von und aktive Auseinandersetzung mit chirurgischen Techniken im vorklinischen Studienabschnitt einerseits das Interesse der Medizinstudierenden an einem operativen Fachbereich fördert und andererseits das Verständnis und die Lernmotivation für das Fach Anatomie steigert. Dabei wurde der Schwerpunkt bewusst auf minimal-invasive chirurgische Techniken gelegt, weil die Laparoskopie – und zunehmend auch die roboterassistierte Chirurgie – bei vielen chirurgischen Eingriffen die favorisierten Operationstechniken sind bzw. sein werden, wenn das hier untersuchte Kollektiv von Medizinstudierenden ärztlich tätig sein wird.

### Frühe und stressfreie Auseinandersetzung mit der Chirurgie steigert das Interesse an diesem Fachgebiet

Das Lehrprojekt führte bei 60 % der Teilnehmenden zu einer Steigerung des Interesses an einer Ausbildung in einem operativen Fachgebiet. Die Kohorten mit „hohem“ und „sehr hohem“ Interesse erhöhten sich von 40 % auf 64 % nach Absolvierung der einzelnen Lehrmodule. Vergleichbare Effekte ließen sich bei amerikanischen Medizinstudierenden durch extrakurrikulare (Wochenend‑)Kurse zum Training chirurgischer Kompetenzen erzielen: So führten diese bei 45 % [24] bis 88 % [19] der Studierenden zu einem gesteigerten Interesse an einer chirurgischen Weiterbildung. Im Gegensatz zu logistisch aufwendigeren Zusatzveranstaltungen bietet die Integration solcher Lehrprojekte in reguläre praktische Lehrveranstaltungen (Präparierkurs) einen niederschwelligen Zugang und erreicht die gesamte Studierendenkohorte – einschließlich derjenigen, die zunächst kein hohes Interesse an chirurgischen Fertigkeiten und Tätigkeiten haben.

Die frühzeitige Exposition der Medizinstudierenden mit chirurgischen Techniken und Tätigkeiten in der Vorklinik baut auf den Erkenntnissen von Götz und Osenberg auf, welche ein ausgeprägtes Interesse an der Chirurgie besonders zu Studienbeginn belegen [12, 18]. In Anbetracht dessen, dass das Interesse an der Chirurgie im weiteren Verlauf des Studiums nachlässt, sollte diese anfängliche Begeisterung als strategischer Vorteil für die Gewinnung des chirurgischen Nachwuchses genutzt werden [21]. Diese initiale Aufgeschlossenheit wurde auch in unserer Umfrage belegt, der zufolge 86 % der Medizinstudierenden ein „hohes“ bzw. „sehr hohes“ Interesse angaben, bereits in der Vorklinik chirurgische Techniken zu erlernen. Das initial vorhandene Interesse spiegelte sich auch darin wider, dass 35 % der Teilnehmenden die laparoskopischen Übungen über die regulären Zeitslots hinaus auch außerhalb der Kurszeiten fakultativ nutzten. Darüber hinaus bietet eine frühzeitige Konfrontation mit der chirurgischen Praxis die Chance, gleich zu Studienbeginn unbegründete Zweifel an den eigenen handwerklichen Fähigkeiten und damit verbundene Vorbehalte gegenüber operativen Fächern abzubauen und spätere Fachentscheidungen aufgrund positiver Vorerfahrungen zu treffen [19].

Neben dem frühzeitigen Aufgreifen des Interesses an chirurgischer Tätigkeit scheint auch die spielerische und ungezwungene Herangehensweise an chirurgische Techniken ausschlaggebend zu sein. Dieser Ansatz wurde in Freitextkommentaren häufig positiv hervorgehoben und könnte eine effiziente Strategie sein, um einige der von Seifert identifizierten Gründe für ein abnehmendes Interesse an chirurgischen Fachrichtungen, wie z. B. fehlende Unterstützung und Wertschätzung durch motivierte Ärztinnen und Ärzte, zu adressieren [22]. Auch Neuhaus hebt hervor, dass Medizinstudierende v. a. durch eine stress- und druckfreie Lernatmosphäre für eine chirurgische Weiterbildung motiviert werden können, um Ängste abzubauen und Sympathie für chirurgische Fachbereiche zu wecken [17].

Andererseits führte eine frühzeitige Konfrontation mit chirurgischen Arbeitstechniken auch dazu, dass es bei einem – zwar vergleichsweise kleinen – Studierendenkollektiv zu einem Interessenrückgang an einer späteren operativen Tätigkeit kam. Diese Diversifizierung der Interessenlagen zu Studienbeginn deutet auf eine realistische Selbsteinschätzung der Studierenden hin und kann durchaus auch hilfreich sein für eine talentorientierte spätere Fachgebietsentscheidung.

### Frauen und Männer sind gleichermaßen an der Chirurgie interessiert

Die Befragung hat ergeben, dass das Geschlecht keinen signifikanten Einfluss auf das Interesse an einer chirurgischen Tätigkeit hat. Die tradierte Annahme, es seien überwiegend Männer an der Chirurgie interessiert, ließ sich – zumindest am Anfang des Medizinstudiums – nicht bestätigen. Diese Erkenntnis ist besonders relevant vor dem Hintergrund, dass der Frauenanteil im Medizinstudium in Deutschland derzeit bei 62 % – in unserem untersuchten Kollektiv 67,5 % – liegt und somit eine zunehmende Feminisierung der Medizin zu beobachten ist [6]. Da Frauen jedoch seltener als Männer das Ziel haben, sich in einem chirurgischen Fach zu spezialisieren [10, 16], wird die Chirurgie – zusätzlich zum allgemeinen demographischen Wandel – vor große Nachwuchsprobleme gestellt [13]. In einer Studie des Royal College in Ireland wurde als Ursache herausgearbeitet, dass sich weibliche Studierende bei der späteren Berufswahl v. a. durch die Arbeitsbedingungen beeinflussen lassen [9]. Es darf somit nicht nur das Ziel sein, das fachliche Interesse der Medizinstudierenden an der Chirurgie zu erhöhen, sondern auch die Arbeitsbedingungen in den operativen Fächern attraktiver zu gestalten [10] – z. B. durch Ermöglichung von Teilzeitbeschäftigung, flexible Arbeitszeiten und planbare Ausbildungskurrikula [14, 15].

### Vorangegangene Ausbildung beeinflusst das Interesse für die Chirurgie

Das Lehrprojekt führte zwar auch bei Medizinstudierenden mit vorheriger Ausbildung oder Studium zu einem gesteigerten Interesse an einer späteren chirurgischen Tätigkeit, jedoch signifikant weniger ausgeprägt als bei Medizinstudierenden ohne entsprechende Vorbildung. Eine mögliche Erklärung könnte sein, dass die überwiegend im Gesundheitssektor absolvierten Ausbildungen mit Erfahrungen verbunden waren, die zu Vorbehalten gegenüber chirurgischen Fächern geführt hatten. Dies legt auch eine Studie nahe, die einen Rückgang des Interesses der Studierenden an einer chirurgischen Weiterbildung im Verlauf des Medizinstudiums beobachtete, welcher mutmaßlich auf die Erfahrungen mit den Arbeitsbedingungen im chirurgischen Bereich zurückgeführt wurde [16].

### Praktische chirurgische Bezüge steigern die Lernmotivation für die Anatomie

Die Verknüpfung von theoretischen anatomischen Lerninhalten mit deren praktischer Anwendung im chirurgischen Kontext führte bei über der Hälfte der Medizinstudierenden zu einem gesteigerten Interesse am Fach Anatomie (59 %) und insbesondere zur vermehrten Beschäftigung mit den topographischen Aspekten (56 %). Als einen Hauptgrund nannten die Teilnehmenden das durch die praktischen Übungen geweckte Bewusstsein für die klinisch-chirurgische Relevanz der Anatomie – was sich mit den Ergebnissen einer Studie deckt, in der Medizinstudierenden die Laparoskopie ebenfalls an einem Körperspender vorgeführt wurde [11]. In Freitextkommentaren wurde insbesondere geäußert, dass das laparoskopische Training dazu inspiriert habe, die Anatomie aus verschiedenen Blickwinkeln, Perspektiven und in Bezug zu chirurgischen Prozeduren zu betrachten – ein Ansatz, der über das klassische Anatomielehrbuch- und Atlaskonzept hinausgeht. Vergleichbare Steigerungen der Lernmotivation und des Verständnisses anatomischer Zusammenhänge ließen sich auch durch aktive Teilnahme von Medizinstudierenden des vorklinischen Studienabschnittes an einem interdisziplinären Kongress für minimal-invasive Chirurgie erzielen [3].

### Ausblick

Zum einen konnte das Lehrprojekt belegen, dass die Integration praxisnaher chirurgischer Techniken in den vorklinischen Anatomieunterricht ein gesteigertes Interesse an einer späteren chirurgischen Tätigkeit weckt. Zum anderen konnte die Lernmotivation für das Fach Anatomie und das Verständnis für deren klinische Relevanz erhöht werden. Die durch die drei Module (Klinik-Vorlesung, Laparoskopie am Körperspender, laparoskopische Übungen) erzielten didaktischen Effekte und die Interessensteigerung für eine spätere berufliche Laufbahn in der Chirurgie geben Anlass, dieses Lehrprojekt in das Kurrikulum dauerhaft zu integrieren und auch an anderen medizinischen Hochschulen zu implementieren – zumal der personelle und technische Ressourcenbedarf mit Unterstützung seitens der operativ tätigen Kliniken und der Medizintechnik relativ gut zu bewältigen ist.

Allerdings sollten longitudinale Untersuchungen überprüfen, ob das in dieser Querschnittsanalyse gesteigerte Interesse der Medizinstudierenden an einer chirurgischen Tätigkeit langfristig Bestand hat und tatsächlich zum beruflichen Einstieg in ein chirurgisches Fachgebiet führt. Hoffnungsvoll stimmt in diesem Zusammenhang eine Studie des Columbia University College of Physicians and Surgeons, der zufolge sich ein stetiger Rückgang von Bewerbungen für chirurgische Weiterbildungen umkehrte und ein Anstieg der Bewerberzahlen einsetzte, nachdem Medizinstudierende mit verschiedenen chirurgischen Lehrprogrammen gezielt gefördert wurden [15].

## Fazit für die Praxis

- Medizinstudierende haben geschlechtsunabhängig ein hohes Interesse, chirurgische Fertigkeiten bereits in der Vorklinik zu erlernen.
- Frühzeitige Einbindung und stressfreies Einüben minimal-invasiver chirurgischer Techniken in der Vorklinik fördern das Interesse für eine chirurgische Laufbahn.
- Integration von chirurgischen Arbeitsmethoden und damit verbundenen klinischen Bezügen in den Anatomieunterricht steigern die Lernmotivation für die makroskopisch-topographische Anatomie.

## Supplementary Information

Fragebogen
