# Supplementary material for: Fostering young talents for surgery: Laparoscopy in preclinical education—Too early or exactly right?
Source: Chirurgie (Heidelb). 2024 Aug 12;96(2):136–44. [Article in German] doi: 10.1007/s00104-024-02145-3 (PMC11785606; doi:10.1007/s00104-024-02145-3)
Supplement: Supplementary file 1 — Fragebogen [file 104_2024_2145_MOESM1_ESM.docx]

**Supplementary Material**

**Tab. S1: Fragebogen**

| **Soziodemographische Daten** | | |
| --- | --- | --- |
| Frage 1) | Bitte geben Sie Ihr Alter an: | Freitext |
| Frage 2) | Bitte geben Sie Ihr Geschlecht an: | Freitext |
| Frage 3) | Haben Sie vor Ihrem Medizinstudium bereits eine Ausbildung oder Studium begonnen oder abgeschlossen? (falls ja, bitte im Freitext präzisieren) | - Nein - ja, folgende/s: Freitext |
| **Nutzung der Laparoskopie-Trainer** | | |
| Frage 4) | Wie lange haben Sie die Laparoskopie-Trainer während des Präparierkurses insgesamt genutzt? | - überhaupt nicht - weniger als 10 Minuten - 10 Minuten bis 30 Minuten - 30 Minuten bis 1 Stunde - länger als 1 Stunde |
| Frage 5) | Falls Sie die Laparoskopie-Trainer „überhaupt nicht“ oder „weniger als 10 Minuten“ genutzt haben, können Sie Gründe benennen? [Mehrfachantworten möglich] | - kein Interesse - keine Zeit wegen des arbeits- und lernintensiven Präparierkurses - zu hohe Nachfrage durch andere Studierende - mir genügte die kurze Zeit, um einen Eindruck zu erhalten - technische Probleme oder Unkenntnis bei der Bedienung - ich hätte eine bessere Anleitung während der Nutzung benötigt  (z. B. Handhabung der Instrumente) - mir fehlte der praktische Bezug oder ein konkretes Ziel - Sonstiges: Freitext |
| Frage 6) | Wie beurteilen Sie den Zeitpunkt, bereits in der Vorklinik einen Einblick in eine minimalinvasive chirurgische Technik durch die Nutzung der Laparoskopie-Trainer zu erhalten? | - Ich halte den Zeitpunkt für sinnvoll - Ich halte den Zeitpunkt für verfrüht - keine Antwort |
| **Relevanz der Laparoskopie-Trainer für die anatomische Lehre** | | |
| Frage 7) | Hat sich Ihr Interesse für die Anatomie durch das Angebot der Laparoskopie-Trainer verändert? | - gestiegen - unverändert - gesunken |
| Frage 8) | Hat die Nutzung der Laparoskopie-Trainer Sie angeregt, sich anders mit der Topografie des Situs zu beschäftigen und so positiv zum Lernerfolg beigetragen? | - ja - nein - keine Antwort |
| Frage 9) | Hat sich Ihr anatomisches Verständnis durch das Angebot der Laparoskopie-Trainer verändert? | - gestiegen - unverändert - gesunken |
| **Relevanz der Laparoskopie-Trainer für das Interesse an einem operativen Fachbereich** | | |
| Frage 10) | Wie hoch ist Ihr Interesse, bereits während des vorklinischen Studienabschnittes operative Fertigkeiten zu erlernen? | - sehr hoch - hoch - mittel - gering - sehr gering |
| Frage 11) | Hat sich Ihr Interesse, sich in einem operativen Fach ausbilden zu lassen, durch das Angebot der Laparoskopie-Trainer verändert? | - gestiegen - unverändert - gesunken |
| Frage 12) | Wie hoch war Ihr Interesse VOR dem Präparierkurs sowie dem Angebot der Nutzung der Laparoskopie-Trainer, sich in einem operativen Fach (Chirurgie, Gynäkologie, Urologie, etc.) ausbilden zu lassen? | - sehr hoch - hoch - mittel - gering - sehr gering |
| Frage 13) | Wie hoch ist Ihr Interesse JETZT, nach Abschluss der ersten beiden Abschnitte des Präparierkurses sowie dem Angebot der Nutzung der Laparoskopie-Trainer, sich in einem operativen Fach ausbilden zu lassen? | - sehr hoch - hoch - mittel - gering - sehr gering |
| Frage 14) | Sollen wir auch in den kommenden Präparierkursen die Nutzung der Laparoskopie-Trainer anbieten? | - ja - unentschlossen - nein |
| Frage 15) | Teilen Sie in Freitext-Form Ihre weiteren Kommentare und Einschätzungen mit: | Freitext |
